# A proposal to analyze the progression of non-dialytic chronic kidney disease by surrogate endpoints: introducing parametric survival models

**DOI:** 10.3389/fmed.2023.1029165

**Published:** 2023-05-18

**Authors:** Renato Erohildes Ferreira, Helady Sanders-Pinheiro, Fernando Antonio Basile Colugnati

**Affiliations:** ^1^Post-Graduation Program in Health, School of Medicine, Federal University of Juiz de Fora, Juiz de Fora, Brazil; ^2^NIEPEN, Department of Clinics, School of Medicine, Federal University of Juiz de Fora, Juiz de Fora, Brazil; ^3^NIEPEN, Department of Internship, School of Medicine, Federal University of Juiz de Fora, Juiz de Fora, Brazil

**Keywords:** survival analysis, chronic kidney disease, parametric regression models, surrogate endpoint, kidney disease progression

## Abstract

**Introduction:**

Chronic kidney disease (CDK) progression studies increasingly use surrogate endpoints based on the estimated glomerular filtration rate. The clinical characteristics of these endpoints bring new challenges in comparing groups of patients, as traditional Cox models may lead to biased estimates mainly because they do not assume a hazard function.

**Objective:**

This study proposes the use of parametric survival analysis models with the three most commonly used endpoints in nephrology based on a case study. Estimated glomerular filtration rate (eGFR) decay > 5 mL/year, eGFR decline > 30%, and change in CKD stage were evaluated.

**Method:**

The case study is a 5-year retrospective cohort study that enrolled 778 patients in the predialysis stage. Exponential, Weibull, Gompertz, lognormal, and logistic models were compared, and proportional hazard and accelerated failure time (AFT) models were evaluated.

**Results:**

The endpoints had quite different hazard functions, demonstrating the importance of choosing appropriate models for each. AFT models were more suitable for the clinical interpretation of the effects of covariates on these endpoints.

**Conclusion:**

Surrogate endpoints have different hazard distributions over time, which is already recognized by nephrologists. More flexible analysis techniques that capture these relevant clinical characteristics in decision-making should be encouraged and disseminated in nephrology research.

## 1. Introduction

Chronic kidney disease (CKD) is progressive, and its evolution is associated with a complex network of multifactorial interactions among etiology, comorbidities, and behavioral aspects. Treatments aim to preserve renal function, postponing as much as possible the needs of any renal replacement therapy (RRT) (1–3).

Although adequate management and treatment of the underlying clinical conditions effectively delay CKD progression, adverse outcomes remain high, and conflicting results are observed (4, 5). Nevertheless, there is a consensus among nephrologists that early predictions of CKD progression are essential for optimizing clinical management and reducing the burden of associated diseases (5).

Because it is a highly heterogeneous disease with hazards competing for the need for RRT and/or death, it is difficult to distinguish the onset of critical renal function decline from periodic variations in glomerular filtration rate (GFR) measurements (5, 6). Recent studies suggest that 12 months is a critical window of control to reverse the deterioration of CKD associated with diabetes mellitus (DM) after diagnosis of the previous stage (5, 6). However, for CKD of other causes, the ideal time to define rapid decline (3, 6, 24, and 48 months) is controversial (5, 6). Several surrogate endpoints have been proposed as reference endpoints for rapid disease progression: annual decay > 5 mL/min/1.73 m^2^ of estimated GFR (eGFR) (3), 30% loss of eGFR relative to the baseline in up to 24 or 48 months (7–9), or change in CKD stage (3, 5).

The use of surrogate endpoints to evaluate CKD progression in randomized clinical trials has been justified by the possibility of reducing sample sizes and follow-up time. A surrogate endpoint is also expected to predict a treatment’s clinical benefit, damage, or lack thereof, especially at shorter time intervals, which adds important complexity to the data analysis, as seen below (10–14).

Recent studies on the evolution of CKD suggest that the eGFR slope can be a viable surrogate endpoint for clinical trials with large samples (14). However, for observational studies, a predetermined loss of eGFR may be considered an event that occurs as a function of time based on periodic clinical evaluations. In real-life data, these evaluations are scheduled according to the severity of the disease and comorbidities, resulting in varying measurement times among patients.

Therefore, survival analysis (SA) techniques can be applied to evaluate factors that influence the time to occurrence of these endpoints, similar to the analysis of traditional endpoints, such as the need for RRT or death (15). Another issue to consider is that the incidence of these surrogate endpoints is not constant throughout treatment. Alternative and more flexible forms of SA beyond the Cox proportional hazards models should be considered. Parametric models of SA provide such alternatives and flexibility and deserve greater attention in studies of these issues (16, 17).

This article aims to introduce the use of parametric regressions in SA for observational studies of the progression of predialysis CKD. We provide step-by-step instructions for this technique and a critical reference for its application in the field of nephrology. To achieve this goal, we analyze the behaviors of surrogate endpoints that are commonly used to evaluate rapid CKD progression in a case study.

### 1.1. Gentle introduction to survival analysis

SA techniques model the time until the occurrence of a given event.

Consider the random variableT, defined as the time until the occurrence of an endpoint of interest. T follows the probability distributionF(t), which describes the probability of an individual reaching the endpoint by time *t*, i.e., F(t)=P(T<t). Thus, the survival functionS(t) is defined as the probability of an individual surviving for more than a certain time t or for at least one time equal tot and is expressed asS(t)=Pr(T>t)=1−F(t) (18). The choice of an adequate probability distribution is a key point in the parametric modeling of SA, as will be seen below (18–20).

The hazard function,h(t)=f(t)/F(t), is defined as the instantaneous hazard of an individual suffering the endpoint, where f(t) is the function distance derived from F(t). This function can be represented graphically in the form of curves, which are very useful in interpreting the occurrence of the endpoint. These curves are essential for understanding the patterns of the functional form of hazard for different conditions, such as types of treatment or explanatory covariates. In general, the curves should reflect previous clinical knowledge about the progression of the disease/treatment (18, 20).

From a descriptive point of view, nonparametric techniques for SA such as life tables, the Kaplan–Meier (21) and Nelson–Aalen (22, 23) estimators are the most commonly applied. These techniques, available in most statistical packages, have great graphical appeal and allow testing of differences between survival curves, albeit for only one categorical variable (16, 17). The main limitation of this approach is the impossibility of performing multivariate analysis.

The popular proportional hazards model introduced by Cox (24) is a semiparametric model, which has the advantage of not making assumptions about the probability distribution F(t) and consequently the underlying hazards function. The name proportional hazards (PH) comes from the assumption that the hazard ratio (HR) between categories of a variable is constant over time. This assumption is often violated, especially when the follow-up time is long, which reduces the accuracy of assumptions (15–17, 25, 26).

In the parametric approach, the hazard functionh(t) is defined based on a probability distribution assumed according to the empirical experience of the occurrence of events over time in the population under study. Therefore, parametric approaches are more informative and flexible than nonparametric and semiparametric approaches (26, 27)_,_ despite the additional difficulty of choosing the most appropriate probability distribution.

Advantageously, the parametric models can assume two types of parameterization: (1) proportional hazards (PH), the same assumption used in the Cox models, which should be used when the interest is in the average hazard, or (2) accelerated failure time (AFT), if the researcher is interested in the time of occurrence, i.e., if the endpoint will occur earlier or later in a given follow-up period (26, 27). This reparameterization allows a more intuitive clinical interpretation because the parameter measures the effect of the covariate over time until the endpoint, i.e., if there is an acceleration or a delay of the endpoint (26).

The flexibility of parametric models may be more appropriate for evaluating CKD surrogate endpoints and allowing a better understanding of critical levels of renal function decline. However, few methodological studies have comparatively evaluated the adequacy of SA statistical techniques in clinical studies (19).

## 2. Methods

This methodological study proposes a critical evaluation of parametric regression techniques in survival analysis (SA) by applying them to a historical cohort with real data from patients with predialysis CKD. We present interpretations of the main concepts of SA and compare the adequacy and performance of the parametric models for proportional hazards (PH) and accelerated failure time (AFT) using the distributions most commonly found in statistical software: exponential, Weibull, Gompertz, lognormal, and loglogistic. We provide a step-by-step model for parametric survival analysis, and interested parties may request the programs used for the analyses by contacting the corresponding author.

### 2.1. The case study sample

The data used for the proposed modeling are from a retrospective cohort extracted from an electronic records database. The sample used in the study consisted of patients seen at the Hiperdia Center of Juiz de Fora [Secondary Health Care Program created in 2010 by the Health Secretariat of the Government of Minas Gerais for the treatment of hypertension (AH), DM, and CKD from August 2010 to December 2014] (28, 29). The study was approved by the Human Research Ethics Committee of UFJF (CAAE no. 0173.0.180.420-11).

This dataset serves as a case study to demonstrate the step-by-step process of modeling with real-world data. However, our analysis does not intend to advance clinical knowledge. The selection of variables included in the model, as presented, follows commonly used covariates in studies related to the progression of chronic kidney disease (CKD). The data used in this study were not specifically collected for this article but were obtained through ethical and administrative agreements. The inclusion criteria for admission of patients with AH to the Hiperdia Center were as follows: diagnosis of lack of response to concomitant use of three or more antihypertensive drugs prescribed in pharmacologically effective doses, target organ damage or suspicion of secondary arterial hypertension; and for patients diagnosed with DM, type 1 DM or type 2 DM with metabolic control according to the therapeutic goal (28, 29).

The historical cohort was defined as patients aged ≥ 18 years with AH and/or DM who had records of at least two consultations in the predialysis multidisciplinary CKD outpatient clinic (3).

The data were collected by consulting electronic medical records. Demographic information was collected at admission, and the other variables were collected during periodic follow-up visits (28, 29).

### 2.2. Demographic and clinical variables

eGFR was calculated from serum creatinine using the CKD-EPI equation (3). For the purposes of the methodological application, we used the following variables for multivariate adjustment of the models: sex, age (≤ or > 69 years, given the median was 69.5 years), eGFR at the first visit, diagnosis of DM and diagnosis of AH with high hazard for cardiovascular disease (CVD). The risk of CVD was estimated using the Framingham score [≥ 14 points (risk of cardiovascular outcome > 20% in 10 years)] (28, 29).

### 2.3. Endpoints

As surrogate endpoints, we used the *Kidney Disease: Improving Global Outcomes* (KDIGO) proposals as indicators of CKD progression:

- Annual GFR decay > 5 mL/min/1.73 m^2^, calculated as the difference between two measurements multiplied by the proportion of the number of months separating the measurements in the year (months/12) (3);
- Decrease in eGFR of 30% compared with baseline in up to 24 or 48 months (7–9); or
- Change in the CKD stage (3–5).

In our analysis, we only considered the initial occurrence of each endpoint, and as a result, our dataset includes the time of the first measurement when the endpoint is achieved, or the last observation, which serves as a form of censorship.

### 2.4. Survival analysis modeling

In addition to the descriptive analyses, survival models were fit to exponential, Gompertz, Weibull, lognormal and loglogistic distributions in both parameterizations (PH and AFT).

The method for adjusting the models is presented step-by-step to show in a didactic manner important steps that ensure adequate modeling. The syntaxes used to generate the models and graphs presented in this document will be made available in Supplementary material.

In a practical manner, we can summarize the parametric SA in the five main steps presented below. All analyses were performed using STATA 15 software (Data Analysis and Statistical Software, College Station, Texas, United States).

Step 01—define the endpoint and statement of the survival study:

We used the surrogate endpoints to predict CKD progression, as already mentioned. For the SA study statement, we used the time variable in “months” relative to the number of months of patient follow-up from their first consultation (baseline or month zero) until the occurrence of the event or end of the follow-up defined for the study (censorship). The event variable was a decrease > 5 mL/year in eGFR compared with the baseline during the analysis period, i.e., eGFR > 5%, generating a dichotomous variable called “failure” (failure = 1). The same procedure was used for the other two endpoints: 30% drop in eGFR (failure = 1) or change in stage (failure = 1). The time variable should be related to these events. The censorship variable was coded (censorship = 0).

Step 02—general survival function by the Kaplan–Meier (KM) method and general hazard function by the smoothed hazard estimate method:

Graphically estimate the survival function using the KM method. Patients were censored for death, beginning of RRT or end of follow-up (censorship = 0). The nonparametric approach to estimate the hazard function is flexible, modeless and data-driven. No form assumptions are imposed, except that the hazard function is a smooth function, for which we used the smoothed hazard estimate graph for the one already enabled in STATA.

Step 03—estimate the curves for the functions and the parametric models:

The forms of the survival function and hazard function were calculated according to the models available in STATA: exponential, Weibull, loglogistic, lognormal and Gompertz, always adjusting for the CKD-EPI value at baseline. The models can be compared using an estimated fit measure and visual inspection by graphs. At this point, the nephrologist’s view is important because the curves should represent the expected clinical evolution for the endpoint.

Step 04—generate multivariate models for the preselected covariates as being explanatory for the eGFR decay:

The eGFR at baseline and the comorbidities DM and AH were fit as explanatory variables only for illustrative purposes of comparison between the models. The first type of model was for PH, and the expressed parameter was HR. Values >1 indicate an increased hazard of eGFR decay, an increased hazard of a 30% decrease in eGFR, or an increased hazard of changing stages. The second type of model was for AFT, which had a reverse interpretation in which values <1 indicate that the three endpoints may occur earlier.

Step 05—compare the fits of the models by the graphical method:

Several methods of graphical adjustment have been proposed, such as those suggested by Allison (30), which analyze the adequacy of the generalized gamma model based on the diagnostic graphs, that is, through the analysis of residuals. Several types of residuals have been proposed for survival models, and the most frequently reported alternative is the use of generalized Cox-Snell residuals.

## 3. Results

The sample consisted of 778 individuals, most of whom were female (51.6%). The mean age was 68.7 ± 11.8 years and ranged from 20 to 97 years. The mean eGFR was 35.8 ± 12.6 mL/min, with a similar distribution in stages 3A (26.3%), 3B (39.7%) and 4 (27.5%) and less than 10% in stage 5. The follow-up period was 60 months. The prevalence of DM was 29.0% (*n* = 226), and patients with AH were diagnosed with a high risk of CVD (29.8%, *n* = 232; Table 1).

**Table 1:** Demographic and clinical characteristics of the sample at baseline and in relation to the three surrogate endpoints.

Variables		Totals	Mean ± SD	Median	Mean ± SD (*n*)			
					Stage 3A	Stage 3B	Stage 4	Stage 5
Age		778	68.7 ± 11.9	69	63.7 ± 9.1 (214)	69.3 ± 12.0 (309)	72.7 ± 12.4 (205)	69.7 ± 11.4 (50)
eGFR at baseline		778	35.9 ± 12.6	36,9	50.7 ± 3.7 (214)	37.8 ± 4.3 (309)	23.6 ± 4.3 (205)	10.6 ± 3.7 (50)
Prevalences		% (*n*)	Prevalences at each CKD stage % (*n*)					
CKD stage	3A	27.5 (214)						
	3B	39.7 (309)						
	4	26.3 (205)						
	5	6.4 (50)						
Age	Up to 69 years	49.5 (393)			75.2 (161)	45.9 (142)	33.2 (68)	44.0 (22)
	>69 years	50.5 (385)			24.7 (53)	54.0 (167)	66.8 (137)	56.0 (28)
Sex	Female	51.2 (398)			52.8 (161)	56.0 (173)	45.4 (93)	38.0 (19)
	Male	48.2 (380)			47.2 (101)	44.0 (136)	54.6 (112)	62.0 (31)
DM		29.1 (226)			42.1 (90)	28.5 (88)	19.0 (39)	18.0 (9)
High risk of CVD		29.8 (232)			41.2 (88)	32.7 (101)	76.6 (36)	14.0 (7)
Decay > 5 mL/year of eGFR	Censorship	51.0 (397)			52.3 (112)	50.2 (155)	47.3 (97)	66.0 (33)
	Endpoint	49.0 (381)			47.7 (102)	49.8 (154)	52.7 (108)	34.0 (17)
Decay of 30% of eGFR	Censorship	84.0 (654)			90.2 (193)	87.4 (270)	75.1 (154)	70.0 (37)
	Endpoint	16.0 (124)			9.8 (21)	12.6 (39)	24.9 (51)	26.0 (13)
Change in CKD stage	Censorship	75.7 (637)			62.7 (146)	77.5 (262)	81.4 (179)	100.0 (50)
	Endpoint	24.3 (204)			37.3 (87)	22.5 (76)	18.6 (41)	0.00 (0)

CVD, cardiovascular disease; DM, diabetes mellitus; eGFT, glomerular filtration rate estimated by CKD-EPI; CKD, chronic kidney disease; CKD-EPI, Chronic Kidney Disease Epidemiology Collaboration; SD, Standard deviation.

Regarding progression to CKD, the prevalence of endpoints was as follows: (I) eGFR decay > 5 mL/year: 381 failures (49.0%), (II) eGFR decay >30%: 124 failures (16.0%) and (III) change in CKD stage: 204 failures (24.3%).

In terms of the time until the occurrence of each event, for eGFR decay > 5 mL/year, approximately 60.9% of the failures occurred within 6 months and 26.5% within 12 months. For eGFR decay >30%, most failures (56.4%) occurred after 12 months. For patients with a change in CKD stage, the vast majority of stage changes occurred within 12 months (67.8%; Table 2).

**Table 2:** Occurrence of the surrogate endpoints for CKD progression over follow-up time intervals.

Endpoint	*N*	%
**Decay > 5 mL/year of eGFR**		
Up to 6 months	232	60.8
6 to 12 months	101	26.5
12 to 24 months	44	11.5
>24 months	4	1.05
Totals	381	100.0
**Decay of 30% of eGFR**		
Up to 6 months	18	14.5
6 to 12 months	36	29.0
12 to 24 months	53	42.7
>24 months	17	13.7
Totals	124	100.0
**Change in CKD stage**		
Up to 6 months	75	36.8
6 to 12 months	63	30.8
12 to 24 months	58	28.4
>24 months	8	3.9
Totals	204	100.0

*N*, absolute frequency; %, relative frequency; eGFR, glomerular filtration rate estimated by CKD-EPI; CKD, chronic kidney disease; CKD-EPI, Chronic Kidney Disease Epidemiology Collaboration.

**Figure 1:**
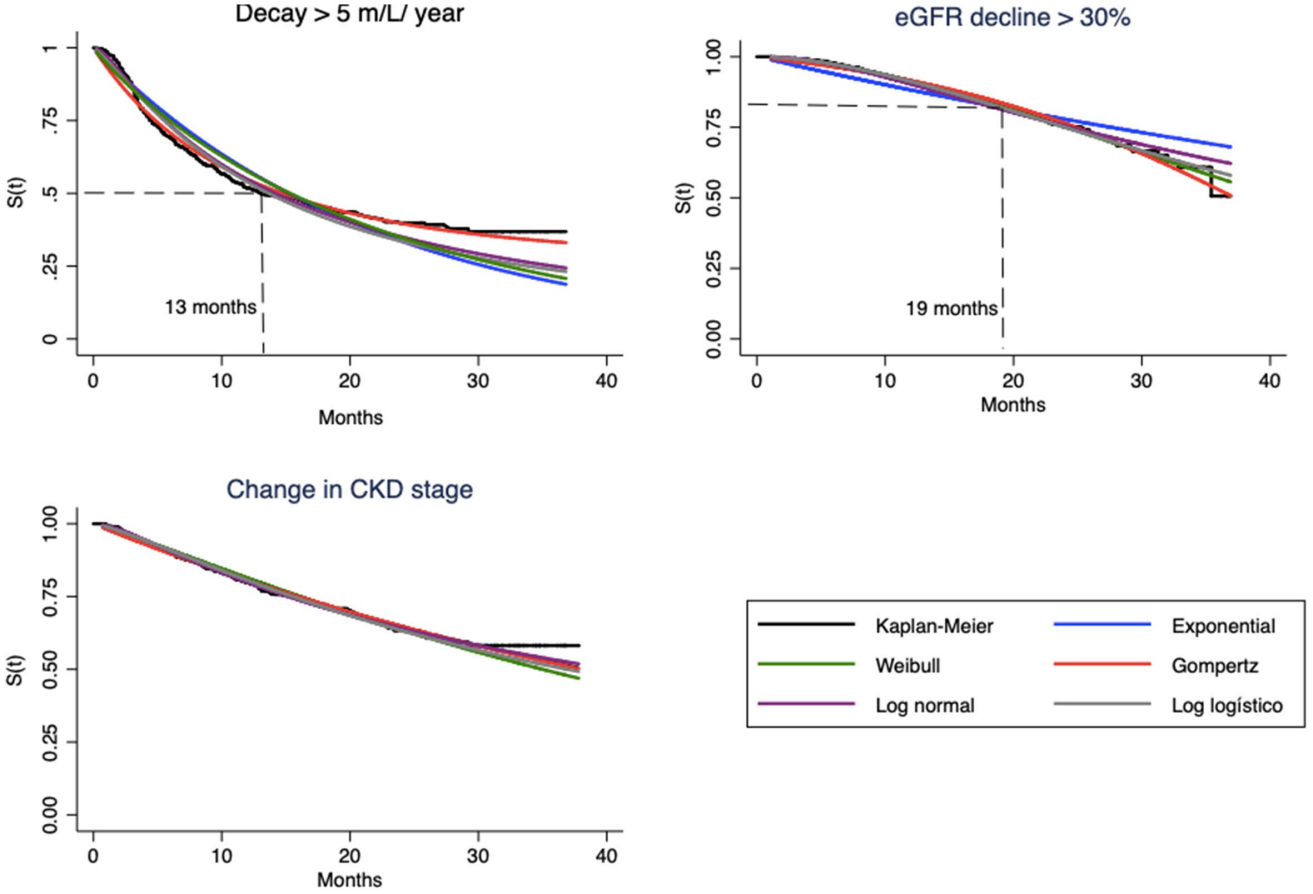
Survival curves estimated by Kaplan–Meier method and parametric model based, for each endpoint.

**Figure 2:**
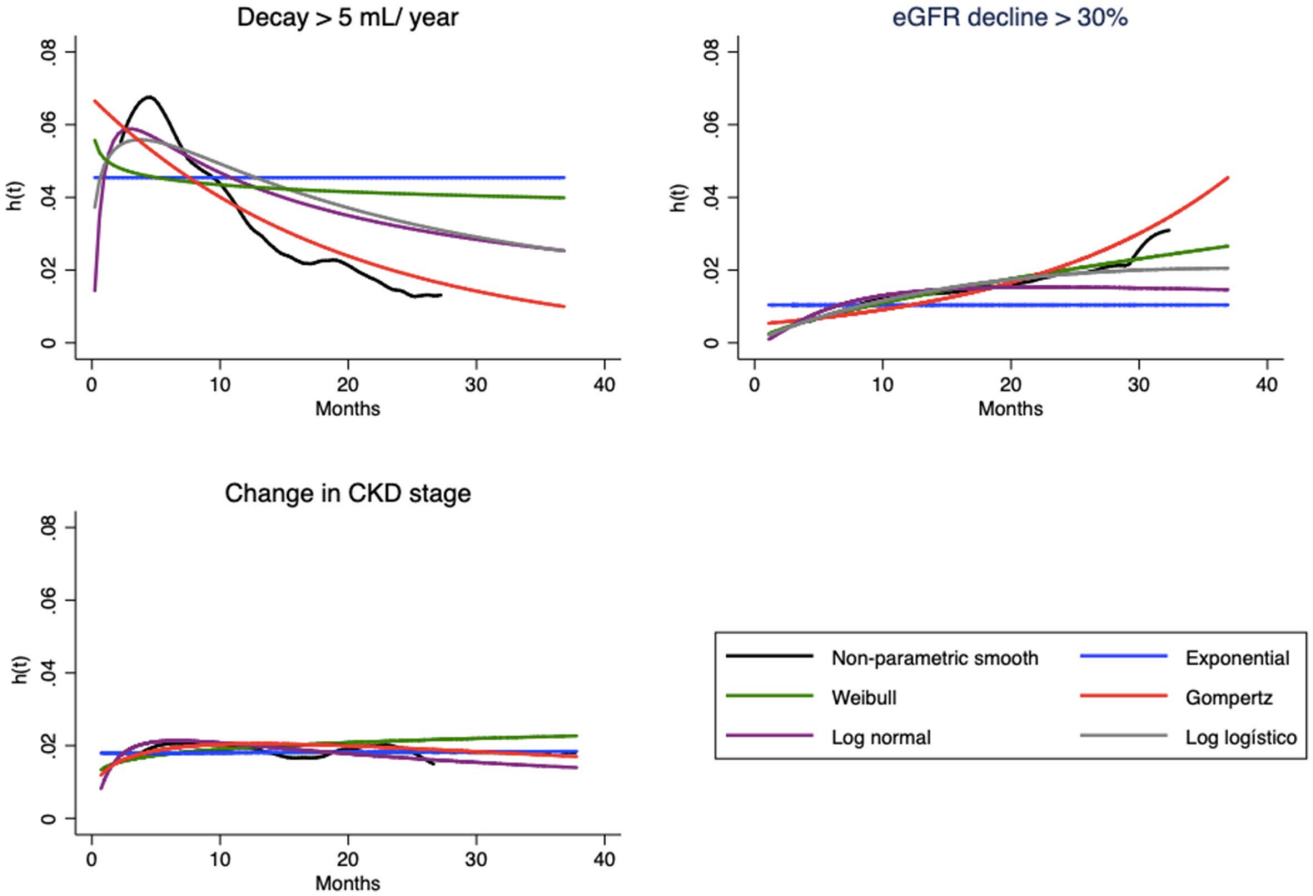
Hazard functions [h(t)] estimated by empirical smooth method and parametric model based, for each endpoint.

This can be understood as a first approach to the hazard function h(t). Decay > 30% has a nonmonotonic characteristic, with peak occurrence between 12 and 24 months, while the other endpoints decay monotonically over time (Table 2). The endpoint of the study was defined as described in “Step 1.” Figures 1, 2 correspond to the curves of Steps 1 and 2 (syntax on Supplementary material). The survival estimates of the parametric models are contrasted with those calculated using the nonparametric Kaplan–Meier and smoothed hazard estimate methods.

Visual evaluation of the graphs for eGFR decay > 5 mL/year shows that the best superposition of the survival curves with the Kaplan–Meier curve indicates almost perfect fits of the Gompertz model (Figure 1) and the hazard function curve (Figure 2). Regarding eGFR decay > 30%, the best fits for the S(t) function were the Gompertz, Weibull and loglogistic distributions (Figure 1). In the case of change in stage, the behavior of the curve was adequately captured by nearly all models, which can be explained by the practically linear trend of the survival function, which facilitated the independent adjustment of the model type (a line is always captured by any model). This behavior only changes after month 30, when no more events occur (Figure 1).

The hazard function h(t), as previously mentioned, should represent the evolution of hazard from a clinical point of view. Note that the shapes of the curves vary according to the type of endpoint (Figure 2). In the case of eGFR loss of 5 mL/year, the tendency is for the instantaneous hazard to decrease as a function of time; i.e., events tend to occur with greater probability at the beginning of treatment. The opposite relationship is observed for eGFR decrease > 30%, with an increase in hazard over time. In both cases, the curves of the Gompertz model are again similar to the empirical curve; the loglogistic curve fits well for the annual drop, and the Weibull curve fits well for the 30% loss. For change in stage, this hazard is apparently constant, which allows the use of any model, including the simplest, such as the exponential model.

Three covariates were used for multivariate fit with the PH and AFT models: eGFR (expressed by CKD-EPI at baseline), diagnosis of DM and AH with high hazard of CVD. Table 3 shows the results for the three endpoints in the different types of models. In grayscale, the best models for each endpoint stand out according to the visual inspection of survival curves and hazard functions, in addition to the analysis of generalized Cox-Snell residuals.

**Table 3:** Multivariate parametric survival models for each surrogate endpoint.

Endpoints, parametrization and estimated parameter		Parametric models										
		Adjusted variables	Exponential		Weibull		Log-normal		Log-logistic		Gompertz	
			Parameter 95% CI		Parameter 95% CI		Parameter 95% CI		Parameter 95% CI		Parameter 95% CI	
**Decay > 5 mL/year of eGFR**												
PH	HR	CKD-EPI base	0.997	0.989–1.006	0.997	0.989–1.006	0.992	0.983–1.001	0.993	0.984–0.997^*^	0.997	0.989–1.005
		DM	1.377	1.105–1.717^*^	1.369	1.099–1.707^*^	1.423	1.121–1.807^*^	1.439	1.127–1.836^*^	1.341	1.076–1.670^*^
		High risk CVD	1.117	0.892–1.400	1.114	0.890–1.396	1.107	0.870–1.409	1.113	0.869–1.424	1.101	0.880–1.378
AFT	TR	CKD-EPI base	1.002	0.993–1.010	1.001	0.993–1.011	1.007	0.998–1.016	1.006	0.997–1.015	-	-
		DM	0.725	0.582–0.904^*^	0.715	0.565–1.904	0.702	0.553–0.891	0.694	0.544–0.887	-	-
		High Risk CVD	0.894	0.714–1.120	0.890	0.700–1.132	0.902	1.709–1.142	0.892	0.704–1.149	-	-
**Decay of 30% of eGFR**												
PH	HR	CKD-EPI base	0.973	0.959–0.987^*^	0.973	0.959–0.987^*^	0.982	0.972–0.992^*^	0.983	0.974–0.992^*^	0.973	0.959–0.987^*^
		DM	0.589	0.402–0.864^*^	0.553	0.363–0.782^*^	0.673	0.523–0.866^*^	0.696	0.552–0.878^*^	0.535	0.365–0.785^*^
		High Risk CVD	0.661	0.459–0.953^*^	0.603	0.418–0.870^*^	0.749	0.588–0.957*	0.746	0.597–0.933^*^	0.601	0.416–0.868^*^
AFT	TR	CKD-EPI base	1.027	1.013–1.042^*^	1.015	1.007–1.024^*^	1.108	1.008–1.028^*^	1.017	1.008–1.026^*^	-	-
		DM	1.695	1.157–2.485^*^	1.439	1.150–1.799^*^	1.485	1.154–1.912^*^	1.436	1.139–1.812^*^	-	-
		High Risk CVD	1.511	1.049–2.176^*^	1.339	1.082–1.658^*^	1.334	1.044–1.704^*^	1.339	1.071–1.673^*^	-	-
**Change in CKD stage**												
PH	HR	CKD-EPI base	1.009	0.997–1.022	1.010	0.998–1.022	1.008	0.997–1.021	1.009	0.998–1.020	1.009	0.997–1.022
		DM	1.429	1.064–1.918^*^	1.430	1.065–1.921^*^	1.458	1.091–1.952^*^	1.439	1.089–1.901^*^	1.429	1.064–1.919^*^
		High Risk CVD	1.308	0.966–1.771	1.307	0.965–1.771	1.252	0.929–1.687	1.248	0.939–1.659	1.308	0.966–1.772
AFT	TR	CKD-EPI base	0.990	0.978–1.002	0.990	0.980–1.001	0.991	0.979–1.002	0.990	0.979–1.001	-	-
		DM	0.699	0.521–0.939	0.730	0.562–0.948	0.652	0.512–0.916	0.694	0.535–0.917	-	-
		High Risk CVD	0.764	0.564–1.034	0.790	0.604–1.033	0.798	0.592–1.075	0.800	0.602–1.064	-	-

Gray shades highlight the best models for each endpoint. PH, Proportional hazard; AFT, Accelerated failure time; HR, Hazard ratio; 95% CI, 95% Confidence Interval; *value of *p* < 0.05. base (baseline).

Note that in some situations, the estimated parameters are different. Taking the estimates for the DM, for the first endpoint, the HR ranges from 1.34 to 1.44 (for the Gompertz and loglogistic models, respectively), while TR varies much less, between 0.69 and 0.74. The parameters of the second endpoint are in the opposite direction. For example, diabetic patients have a 55% mean risk of eGFR loss >30% according to the Weibull model and a 44% longer time for this occurrence. For the other endpoints, the effects are increased hazard and decreased time. This fact can be explained by the data in Table 1; there is a greater share of diabetic patients in the early stages of CKD, and the occurrence of diabetes increases with CKD severity. The opposite pattern is observed for the other endpoints.

The AFT parameterization for the Gompertz model, which showed a good fit for all endpoints, presents mathematical challenges that are only circumventable through intensive computational methods and cannot be implemented in most statistical software, including STATA; consequently, it was not performed.

Thus, Table 4 presents a summary of the interpretations of HR and TR. For this purpose, we use as an example the Weibull model, which presented the second-best fit for the three endpoints based on the visual inspection of the curves of the S(t) and h(t) functions and the interpretation of the DM effect in the occurrence of endpoints.

**Table 4:** Clinical interpretation of the parameters of the Weibull model for the effect of diabetes mellitus in the PH and AFT models for the surrogate endpoints of CKD progression.

Type of model	Parameter	Parameter estimates		Clinical interpretation
**Decay > 5 mL/year of eGFR**				
PH	HR	1.369	>1	Diabetic patients have a 36.9% higher hazard of eGFR decay > 5 mL/year compared with nondiabetic patients.
AFT	TR	0.715	<1	Diabetic patients may have an eGFR decay > 5 mL/year at a time 28.5% earlier than nondiabetic patients. The occurrence of the event is therefore 3.7 months earlier than predicted.
**Decay of 30% of eGFR**				
PH	HR	0.554	<1	Diabetic patients have a 44.6% lower hazard of GFR decay > 5 mL/year than nondiabetic patients.
AFT	TR	1.439	>1	Diabetic patients may have an eGFR decay > 5 mL/year at a time 43.9% later than nondiabetic patients. The occurrence of the event is therefore 8.5 months later than predicted.
**Change in CKD stage**				
PH	HR	1.430	>1	Diabetic patients have a 43% higher hazard of GFR decay > 5 mL/year than nondiabetic patients.
AFT	TR	0.730	<1	Diabetic patients may have an eGFR decay > 5 mL/year at a time 27.0% earlier than nondiabetic patients. The occurrence of the event is therefore 5.1 months earlier than expected.

PH, proportional hazards; AFT, accelerated failure time; HR, hazard ratio; TR, time ratio; 95% CI, confidence interval; eGFR, estimated glomerular filtration rate. For the examples with AFT models, the median survival of the respective endpoints was considered as described in Figure 1.

After performing the multivariate adjustments, we must verify the quality of the fit of each parametric model before selecting a model, which is Step 5.

**Figure 3:**
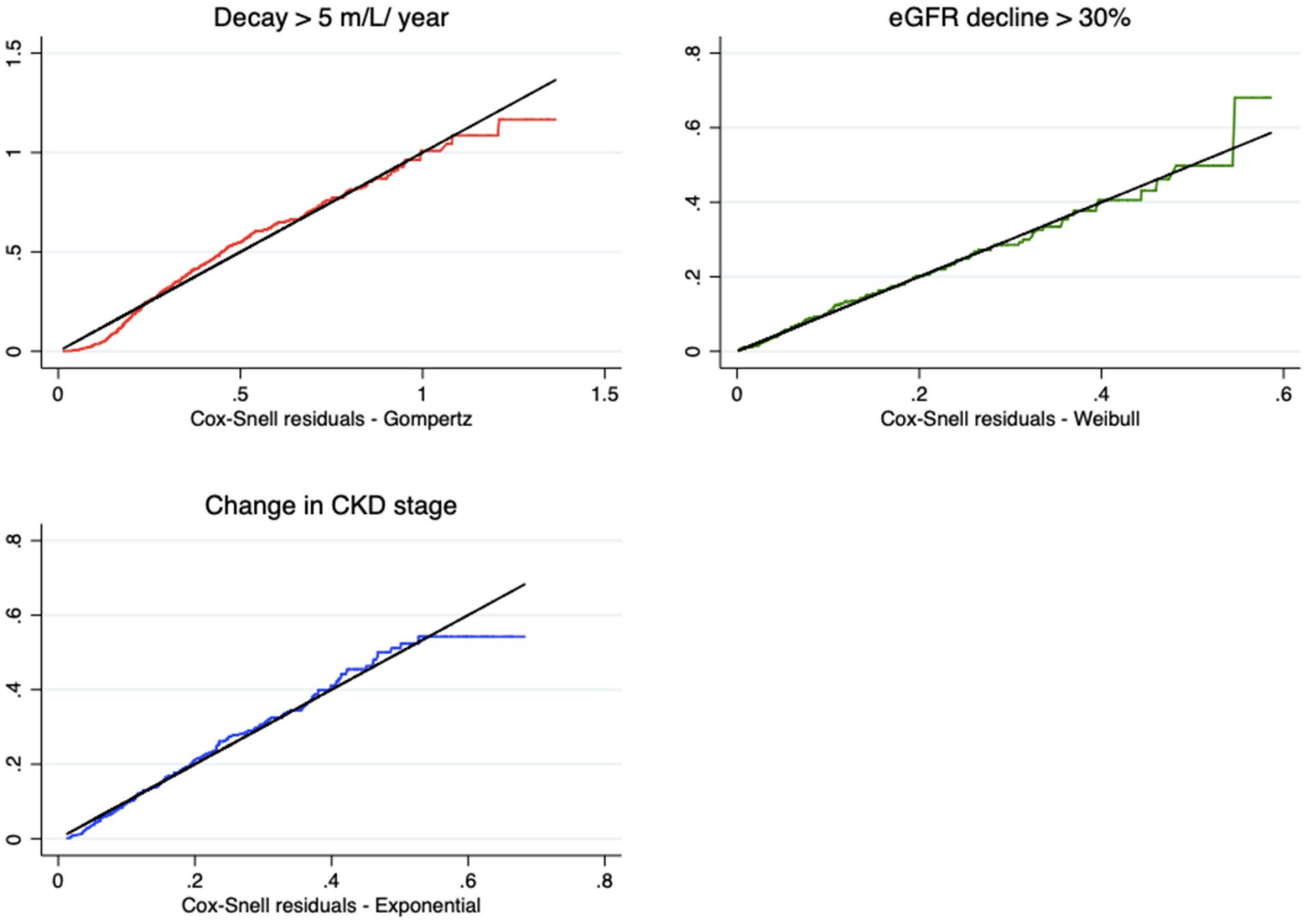
Cox-Snell residuals for the best models for each endpoint.

Most of the techniques proposed for this purpose are graphical and residual analyses. For each model and endpoint, we calculated the values of the Cox-Snell residuals to measure the adequacy of the adjustments. Figure 3 shows the models chosen as the best fit. A good fit of the models is observed, given that the residuals overlap on the reference line of the quartiles of the respective distributions. The residuals move slightly away from the line only near the end of the follow-up time, without compromising the adjustment.

## 4. Discussion

We present the methodology and apply parametric survival models for surrogate endpoints of CKD progression, using a real-world dataset from a historical cohort of patients with CKD undergoing predialysis treatment as a practical example. Despite the increasing use of SA in clinical research in recent decades, interpretations are mostly limited to HRs estimated by Cox semiparametric models. These models, despite answering many research questions, fall short in some situations and may limit the scope of important clinical interpretations of parametric models, as in the case of surrogate endpoints (15).

The use of surrogate endpoints, also termed intermediate endpoints, is increasing and is even defining the design of randomized clinical trials. Consequently, these endpoints will soon cease to be surrogates and will become primary endpoints, which demands a better exploration of this knowledge gap among clinical researchers as a reference for their analyses and interpretations (12–16).

The preference for semiparametric models may reflect the difficulties that researchers in the field often face when dealing with parametric modeling. In parametric models, probability distributions must be assumed and then compared, which requires further technical decisions to be made in a subject that is not typically well-understood by clinical researchers. This complexity may lead to a bias toward simpler semiparametric models, which may have limitations in terms of clinical interpretation, but require less technical expertise (15).

Although the Cox PH model is very flexible, it is not immune to the typical assumptions of statistical modeling. Its fundamental assumption is that the factors under study should have a constant effect on the hazard over time. This assumption is violated for most biological phenomena (31). The assumption of proportionality is violated in almost two-thirds of studies (31). Further, only 36% of studies mentioned the assumption of proportionality, and only 20% mathematically tested this assumption (32).

Patients with CKD undergoing predialysis treatment are routinely compared using absolute or relative measures of treatment effect for primary endpoints such as death and RRT. A limitation of this approach is that its clinical significance depends heavily on the baseline or progression of the patient over time (33). These measures can be very useful when the goal is only to estimate the effect of a covariate at baseline on the final endpoint, that is, whether it increases or decreases the hazard of the patient suffering the endpoint of interest regardless of when it occurs (33, 34). In the case of surrogate endpoints, a complete understanding of how the hazards vary over time may be fundamental for helping the clinician decide the ideal time of interventions to postpone this endpoint. Therefore, understanding whether the endpoint occurs in a shorter or longer time, conditioned on the levels of explanatory or predictive variables, is a key clinical issue for predicting prognosis (35, 36). Therefore, complementary results that allow an understanding of the evolution of treatment as a function of follow-up time, such as the interpretation of survival curves and hazard function, are informative tools to evaluate this evolution (33, 37, 38).

For this reason, we focused in this study on the interpretation of hazard curvesh(t). The hazards may vary over time, and the follow-up times of each individual are usually very different; thus, a more flexible functional form tends to be more reliable than the actual average clinical trajectory of the sample under analysis. It is also possible to use estimates of the survival and hazard curves to construct predictions of expected behaviors (6).

The sample used in the study is similar to the nondialysis CKD populations described by other authors (10–14), particularly the average age, which was close to 70 years. In one-third of CKD patients, the etiology is DM. For patients in the early (3) and intermediate (4, 5) stages, in which the benefit of treatment is potentially greater (5), this representativeness reinforces the hazard estimates.

The differences in modeling on the three types of surrogate endpoints in this study highlights the importance of considering these issues. The hazard curves were markedly distinct. The change of stage endpoint the hazard was constant. As a result, we recommend the use of basic models such as exponential model or even the standard Cox models. For the endpoints defined based on threshold amounts of renal function decay, the hazards exhibit inverse relationship over time. While annual decay > 5 mL monotonically decreases over time, with greater hazards at the beginning of treatment, decay in eGFR greater than 30% showed an increased hazard in later periods. The use of Cox models in this case would fail to capture this information, which has great clinical value, and would probably lead to a biased or incorrect interpretation of the relative hazards (15–17, 25, 26).

We also emphasize that the parametric form of the model can be based entirely on the clinical experience of the researchers. For example, when comparing the predictions of the percentage of patients who remain event-free after a certain follow-up period, the plausibility of the choice of a parametric distribution can be assessed *a priori* by reference to previous relevant studies. If such studies are not available, it will be necessary to rely on the opinions of clinical experts. The opinions of an expert clinician and/or experienced analyst can provide valuable information on the plausibility of certain models and their extrapolations compared with known disease patterns (39).

It is very likely that the results will coincide, at least in the general form of trend and monotonicity, with the empirical estimate of the hazard function, even if only approximately. Hence, the option for a parametric model of SA, followed by the fit analysis of the model chosen by graphical means or information criteria such as the Akaike information criterion (AIC), can bring estimates of effects closer to the clinical reality (39).

Therefore, this study intends to encourage the use of parametric models and serve as a reference for this approach mainly in nephrology. This is perhaps the greatest potential contribution of this study, as it used real data from clinical practice at a CKD treatment center.

However, limitations should be considered. We do not consider any causal model and/or the process of selection of final explanatory/predictor variables for a clinical research statistical model. These issues warrant full discussion in separate articles, and good references are Greenland and Gelman (40, 41). No further modeling aspects were included, such as the inclusion of terms of frailty (individual random effects) or cure fraction (42). The inclusion of these terms in the models may yield completely different results than the present analyses but would require a deeper understanding of these concepts, which is beyond the intended introductory scope of this article. There was also no consideration of establishing an explanatory model for the endpoints studied. The variables, which are well known for their effects among nephrologists, were used as illustrative examples, and one should avoid generalizing these results to clinical practice or even other studies investigating comorbidities such as DM and coronary diseases in patients with CKD.

## 5. Conclusion

Our study aimed to explore the possible applications of parametric survival models in a cohort of CKD patients in the predialysis stage. Although medical research has focused on semiparametric models in recent decades, Violations of the proportionality assumption can lead to biased or inaccurate measurements that may not align with clinical practice. Given the complex nature of CKD and the nuances of clinical practice, the input of a nephrologist is crucial for determining the plausibility of a final model results, and its interpretation. Given the flexibility of the parametric models, by comparing parametric models with relevant studies and expert opinions, we can gain insights into disease patterns and identify potential avenues for future research.

## Data availability statement

The data analyzed in this study is subject to the following licenses/restrictions: the owner did not authorized the publication of the original dataset, even without any identification variable. Requests to access these datasets should be directed to fernando.colugnati@medicina.ufjf.br.

## Ethics statement

The studies involving human participants were reviewed and approved by Comitê de Ética em Pesquisa Humana—UFJF. The patients/participants provided their written informed consent to participate in this study.

## Author contributions

RF: project development, statistical analysis, manuscript writing, and manuscript review. HS-P: writing of the manuscript and critical review of the manuscript. FC: project coordination, statistical analysis, manuscript writing, and manuscript review. All authors contributed to the article and approved the submitted version.

## Funding

This work was supported by Coordenação de Aperfeiçoamento de Pessoal de Nível Superior (CAPES), financial code 001.

## Conflict of interest

The authors declare that the research was conducted in the absence of any commercial or financial relationships that could be construed as a potential conflict of interest.

## Publisher’s note

All claims expressed in this article are solely those of the authors and do not necessarily represent those of their affiliated organizations, or those of the publisher, the editors and the reviewers. Any product that may be evaluated in this article, or claim that may be made by its manufacturer, is not guaranteed or endorsed by the publisher.
