# Supplementary material for: A proposal to analyze the progression of non-dialytic chronic kidney disease by surrogate endpoints: introducing parametric survival models
Source: Front Med (Lausanne). 2023 May 18;10:1029165. doi: 10.3389/fmed.2023.1029165 (PMC10232791; doi:10.3389/fmed.2023.1029165)
Supplement: Supplementary file 1 [file Data_Sheet_1.docx]

Supplementary Material

# Stata sintax for parametric survival modeling

1. ***Load your dataset***

**use** "your_dataset.dta", **clear**

1. ***Declare your survical setings. In this example “months” are the time associated to the event “drc_endpoint”, having 1 as a failure and 0 as censoring***

**stset** months, **failure**(drc_endpoint )

1. ***Adjusting multivariate models. Var1, Var2 e Var3 should be substituted by your variable names***

***MODEL 1 - EXPONENTIAL + Cox-Snell. All Other models follows the same sintax, you just need to chage the distribution in the option** dist()

**streg** Var1 Var2 Var3, **dist**(**exponential**) ***Model estimation**

**predict** surv_exp, **surv * Predict survival values**

**predict** ha_exp, **ha * Predict Hazard values**

**predict** **double** cs_e, **csnell * Predict Cox-Snell residuals**

**sts generate** km_e=s

***MODEL 2 - WEIBULL + Cox-Snell**

streg, dist(weibull)

predict surv_weib, surv

predict ha_weib, ha

predict double cs_w, csnell

***MODEL 3 - LOGNORMAL + Cox-Snell**

streg, dist(lognormal)

predict surv_logn, surv

predict ha_logn, ha

predict double cs_l, csnell

***MODELO 4 - LOGLOGISTIC + Cox-Snell**

streg, dist(loglogistic)

predict surv_loglog, surv

predict ha_loglog, ha

predict double cs_ll, csnell

***MODEL 5 - GOMPERTZ + Cox-Snell**

streg, dist(gompertz)

predict surv_gomp, surv

predict ha_gomp, ha

predict double cs_g, csnell

1. ***Joining all S(t)in one graph, like Figure 1***

**sts graph**, **ci addplot**((**line** surv_exp _t, sort)(line surv_weib _t, sort) (line surv_gomp _t, sort) (line surv_logn _t, sort) (line surv_loglog _t, sort))

1. ***Joining all h(t) in one graph, like Figure 2***

**sts graph** , hazard addplot((line ha_exp _t, sort)(line ha_weib _t, sort) (line ha_gomp _t, sort) (line ha_logn _t, sort) (line ha_loglog _t, sort))

1. ***Generate Cox-Snell residual graphs***

line H_e cs_e cs_e, sort title(Exponencial) legend(off)

line H_w cs_w cs_w, sort title(Weibull) legend(off)

line H_l cs_l cs_l, sort title(Log-normal) legend(off)

line H_ll cs_ll cs_ll, sort title(Loglogistic) legend(off)

line H_g cs_g cs_g, sort title(Gompertz) legend(off)
